# The outcome of IV vitamin C therapy in patients with sepsis or septic shock: a meta-analysis of randomized controlled trials

**DOI:** 10.1186/s13054-023-04392-y

**Published:** 2023-03-13

**Authors:** Baofang Liang, Jianwei Su, Hanquan Shao, Huiying Chen, Baocheng Xie

**Affiliations:** 1 Department of Healthcare-associated Infection Management, Affiliated Dongguan Hospital, Southern Medical University, Dongguan, Guangdong China; 2 Department of Pharmacy, Affiliated Dongguan Hospital, Southern Medical University, Dongguan, Guangdong China; 3 Department of Clinical Pharmacy, Dongguan Tungwah Hospital, Dongguan, Guangdong China; 4 Department of Critical Care Medicine, Affiliated Dongguan Hospital, Southern Medical University, Dongguan, Guangdong China

**Keywords:** Vitamin C, Sepsis, Septic shock, Randomized controlled trials, Mortality, Meta-analysis

## Abstract

**Background:**

To update a meta-analysis of randomized controlled trials (RCTs) and further explore the outcome of IV vitamin C (IVVC) administration in sepsis or septic shock patients.

**Methods:**

This study is a meta-analysis of RCTs. The RCTs of vitamin C therapy in sepsis or septic shock were searched in PubMed, EMBASE and Clinical Trials.gov from inception to January 16, 2023. We registered the protocol with PROSPERO (CRD42022354875). The primary outcome was delta Sequential Organ Failure Assessment (SOFA) score at 72–96 h. Two reviewers independently assessed RCTs according to eligibility criteria: (1) study type: RCT; (2) patient population: patients ≥ 18 years with sepsis or septic shock; (3) intervention: IVVC at any doses as monotherapy or combined with thiamine or and hydrocortisone compared with standard of care, no intervention or placebo (defined as control group); (4) the RCT described short-term mortality or SOFA score. Then, two authors independently extracted related information from RCTs.

**Results:**

Eighteen RCTs (*n* = 3364 patients) were identified in this meta-analysis. There were significant effects in the delta SOFA score from baseline to 72–96 h (MD, − 0.62; 95% CI, − 1.00 to − 0.25; *p* = 0.001) and the duration of vasopressor use (MD, − 15.07; 95% CI, − 21.59 to − 8.55; *p* < 0.00001) with IVVC therapy. Treatment with IVVC was not shown to improve short-term mortality (OR, 0.89; 95% CI, 0.77 to 1.04; *p* = 0.14); nevertheless, dose at 25–100 mg/kg/d subgroup associated with a significant reduction in short-term mortality (OR, 0.80; 95% CI, 0.65 to 0.97; *p* = 0.03). An increase adverse event was observed in IVVC therapy (OR, 1.98; 95% CI, 1.06 to 3.68; *p* = 0.03).

**Conclusion:**

In this meta-analysis, IVVC in sepsis or septic shock patients significantly improved delta SOFA score and reduced the duration of vasopressor use, whereas it was not associated with reduction in short-term mortality and had higher adverse events.

**Supplementary Information:**

The online version contains supplementary material available at 10.1186/s13054-023-04392-y.

## Background

Sepsis is a life-threatening organ dysfunction related to a dysregulated host response to infection [1], and septic shock is a type of sepsis with a higher risk of mortality. This disorder contributes to 11 million deaths worldwide every year [2], which is considered as a primary health threat by the World Health Organization [3]. Despite significant advances in sepsis, no other treatment beyond basic therapy such as source of infection control, fluid resuscitation and vasoactive drugs has sufficient evidence to support to improve mortality [4], and sepsis survivors often suffer from residual organ injury [5]. Consequently, it is very necessary to find effective, safe and economical adjuvant treatments to reduce mortality and financial burden for sepsis.

Vitamin C is a powerful antioxidant drug and a cofactor in the production of numerous biosynthetic enzymes needed for the survival of shock, which participates in the synthesis of intrinsic vasopressin and norepinephrine [6]. Vitamin C cannot be synthesized by the human, and the levels are low in many critically ill patients. As a readily available, inexpensive and few side effects of treatment option, vitamin C supplementation during sepsis has specifically gained increasing interest for years. Early studies confirmed IV vitamin C (IVVC) is associated with decreased inflammatory response and ameliorated outcomes in sepsis [7, 8]. In addition, favorable outcomes in the vitamin C group were reported in some meta-analyses [9, 10]. However, the available evidence remains inconsistent. In another recent meta-analysis, it was reported that IVVC suggested no efficacy in sepsis [11]. The Surviving Sepsis Campaign of 2021: International Guidelines for Management of Sepsis and Septic Shock suggest IVVC for patients with sepsis or septic shock was not recommended, only as a weak recommendation according to the low quality of evidence [12]. Although systematic reviews and meta-analyses discussing IVVC in patients with sepsis were recently published [11, 13, 14], these studies did not include the newer randomized controlled trials (RCTs) [15–17] with a larger population of patients to provide better evidence.

Because of increasing updated trials and inconsistent results in many studies, it is essential to reassess the present evidence about the efficacy and safety of IVVC in sepsis or septic shock patients. The purpose of this meta-analysis of RCTs is to research the impact of IVVC in sepsis patients and carry out subgroup analyses to further explore the efficacy of IVVC with different dose, type of therapy, duration and patient populations.

## Methods

This study performed based on the Preferred Reporting Items for Meta-Analyses (PRISMA) statement [18]. The protocol was prospectively registered in PROSPERO (CRD42022354875).

### Study protocol

We chose RCTs to increase statistical power according to the below criteria: (1) study type: RCT; (2) patient population: patients ≥ 18 years with sepsis or septic shock; (3) intervention: IVVC at any doses as monotherapy or combined with thiamine or and hydrocortisone compared with standard of care, no intervention or placebo (defined as control group); (4) the RCT described short-term mortality or Sequential Organ Failure Assessment (SOFA) score. Conference papers were excluded.

### Literature research and data extraction

We comprehensively conducted search of PubMed, EMBASE and Clinical Trials.gov to identify RCTs using subject terms and uncontrolled terms. The search was last updated on January 16, 2023. Supplemental data for a full list of subject terms and uncontrolled terms were presented the search strategy (Additional file 1: Table S1).

NoteExpress software was utilized to process articles and remove duplicate articles. Two reviewers independently assessed the titles and abstracts to see if they fulfilled the inclusion criteria. When there were disagreements between them, a third-party reviewer was consulted for adjudication to deal with the problems.

The data were retrieved using excel independently by two authors. The relevant information was collected including first author, year of publication, intervention, age, sex, the type of sepsis, SOFA score and so on.

We chose delta SOFA score as primary outcome. Delta SOFA score was defined as calculated by subtracting the SOFA score at enrollment from the corresponding value at 72–96 h. We further extracted the data including different dose (< 25 mg/kg/d, 25–100 mg/kg/d and > 100 mg/kg/d), type of therapy (monotherapy and combined therapy), duration (< 96 h, 96 h and unclear group (defined as such as “study drug infusion was stopped when the last dose was administered or at ICU discharge, study withdrawal, or death”)) and patient populations (sepsis, septic shock and unclear group (defined as indefinable patient population such as sepsis or septic shock patient)) for subgroup analyses to investigate the source of heterogeneity in the effect of IVVC on outcomes as they may exert different treatment effect.

The secondary outcomes were short-term mortality, duration of vasopressor use, vitamin C level and adverse events. The short-term mortality was defined as 28- or 30-day mortality, and when unavailable, hospital mortality was eligible.

### Risk of bias assessment

The Cochrane Risk of Bias tool was used to evaluate the methodological quality of included RCTs to determine the risk of bias independently by two authors [19]. The Cochrane Risk of Bias includes seven areas: random sequence generation, allocation concealment, blinding of participants and personnel, blinding of outcome assessment, incomplete outcome data, selective reporting and other bias. When there were disagreements, a third author was participated in the discussion.

### Statistical analysis

RevMan 5.5 was used for all analyses with the Mantel–Haenszel (M–H) and inverse variance random-effects or fix-effects models for binary and continuous outcomes based on heterogeneity, respectively. Odds ratio (OR) for dichotomous outcomes or mean difference (MD) for continuous outcomes was adopted with 95% confidence intervals (CIs). The medians and interquartile ranges (IQRs) were transformed to means and standard deviation (SD). We evaluated the existence of statistical heterogeneity through the M-H Chi-square test and the inconsistency *(I*^2^) statistic. The heterogeneity of *I*^2^ is not considered statistically significant if it is not exceeded 50%. The heterogeneity has three degrees of low (25%), medium (50%) and high (75%) classified by Guinot et al. [20]. Substantial heterogeneity was identified as *p* < 0.05 or *I*^2^ > 50%. We performed a subgroup analysis focusing on the dose of vitamin C (< 25 mg/kg/d, 25–100 mg/kg/d and > 100 mg/kg/d), vitamin C therapy regimens (monotherapy and combined therapy), vitamin C duration (< 96 h, 96 h and unclear group) and patient populations (sepsis, septic shock and unclear group). Reasons for heterogeneity were planned to explore by conducting sensitivity analyses. To assess publication bias, we conducted funnel plots for outcomes to detect the symmetry of the funnel plots; otherwise, we further used Egger’s test to examine bias. For outcomes with publication bias, we examined the stability using trim and fill analysis and further found out trials with high or unknown risk of bias by Influence Analysis (metaninf). All statistical analyses and assessments of bias risk were conducted by Review RevMan 5.5 and STATA software V12. *p* < 0.05 was defined as statistical significance.

### Certainty of evidence assessment

We utilized the Grading of Recommendations, Assessment, Development and Evaluations (GRADE) [21] methodology to evaluate the quality of evidence. The GRADE encompasses methodological limitations, inconsistency, imprecision, indirectness and publication bias to divide quality of evidence as very low, low, moderate or high from RCTs. The summary of table was generated through the GRADEpro GDT software.

### Trial sequential analysis

Cumulative meta-analysis updating with new RCTs may lead to false positive results (type I error) due to an increased risk of random error from sparse data [22]. Trial sequential analysis (TSA) can reduce the risks of random error because of inadequate sample size or repetitive testing and estimate the required information size (RIS) for meta-analysis [23]. Type I error and power were set as 5% and 80%, respectively. We performed TSA for both delta SOFA score and short-term mortality outcomes by applying TSA version 0.9.5.10 beta software (http://www.ctu.dk/tsa).

## Results

### Eligible studies and study characteristics

**Fig. 1:**
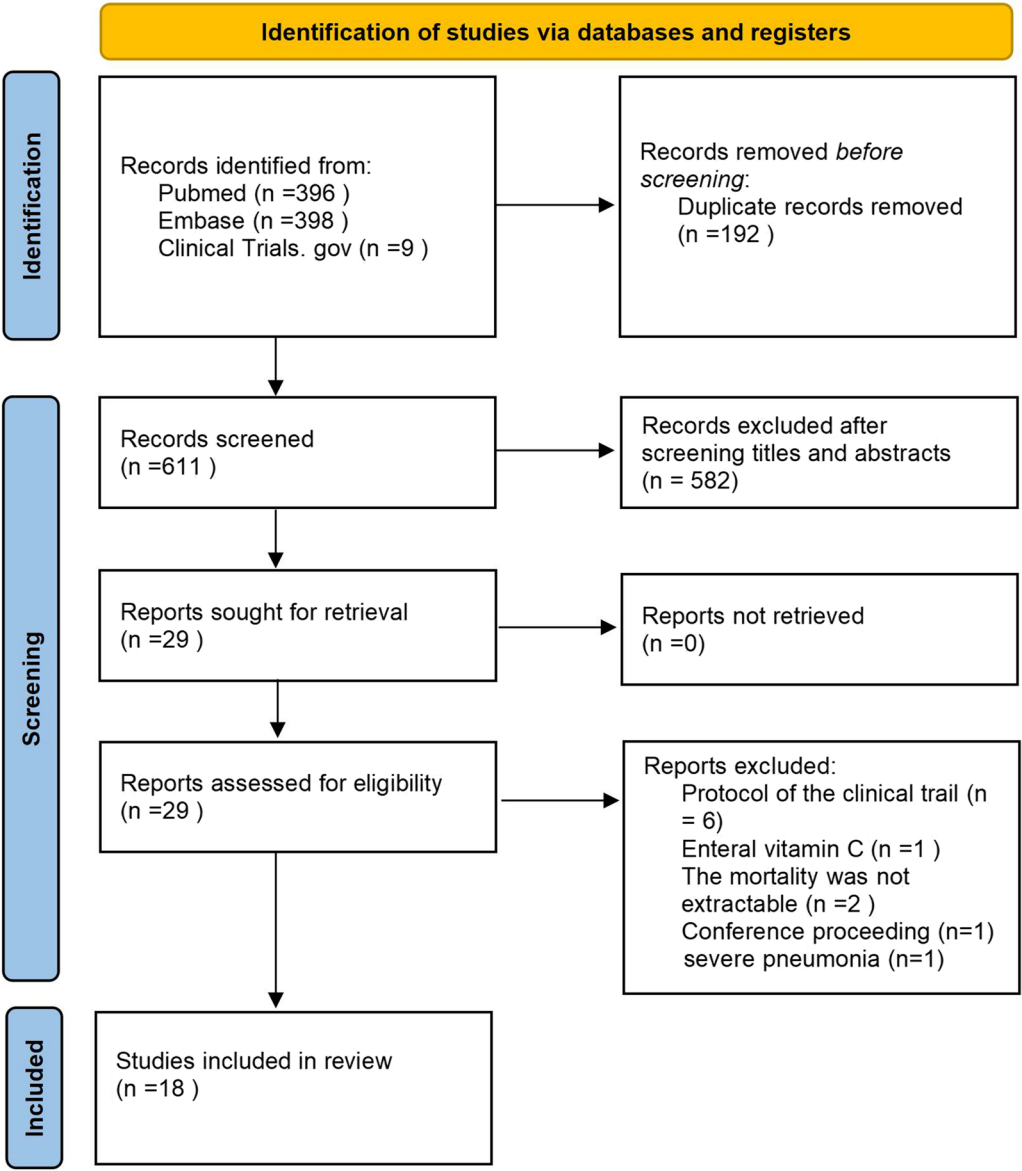
PRISMA 2020 flow diagram for the meta-analysis

We initially identified 803 records, and 192 identical duplicate articles were deleted before screening; 582 studies were excluded by screening titles and abstracts; 11 studies were further removed during the assessment of the full text. Ultimately, 18 eligible RCTs [15–17, 24–38] enrolling 3364 patients were included in this meta-analysis. The PRISMA 2020 flowchart of this study is presented in Fig. 1.

A total of 18 RCTs including 8 multicenter RCT studies and 10 single-center studies were involved with initial vitamin C intervention plan, classification of diseases, initial SOFA score, duration of vasopressors use and mechanical ventilation which were evaluated. The main characteristics are given in Additional file 1: Table S2.

### Risk of bias assessment

**Fig. 2:**
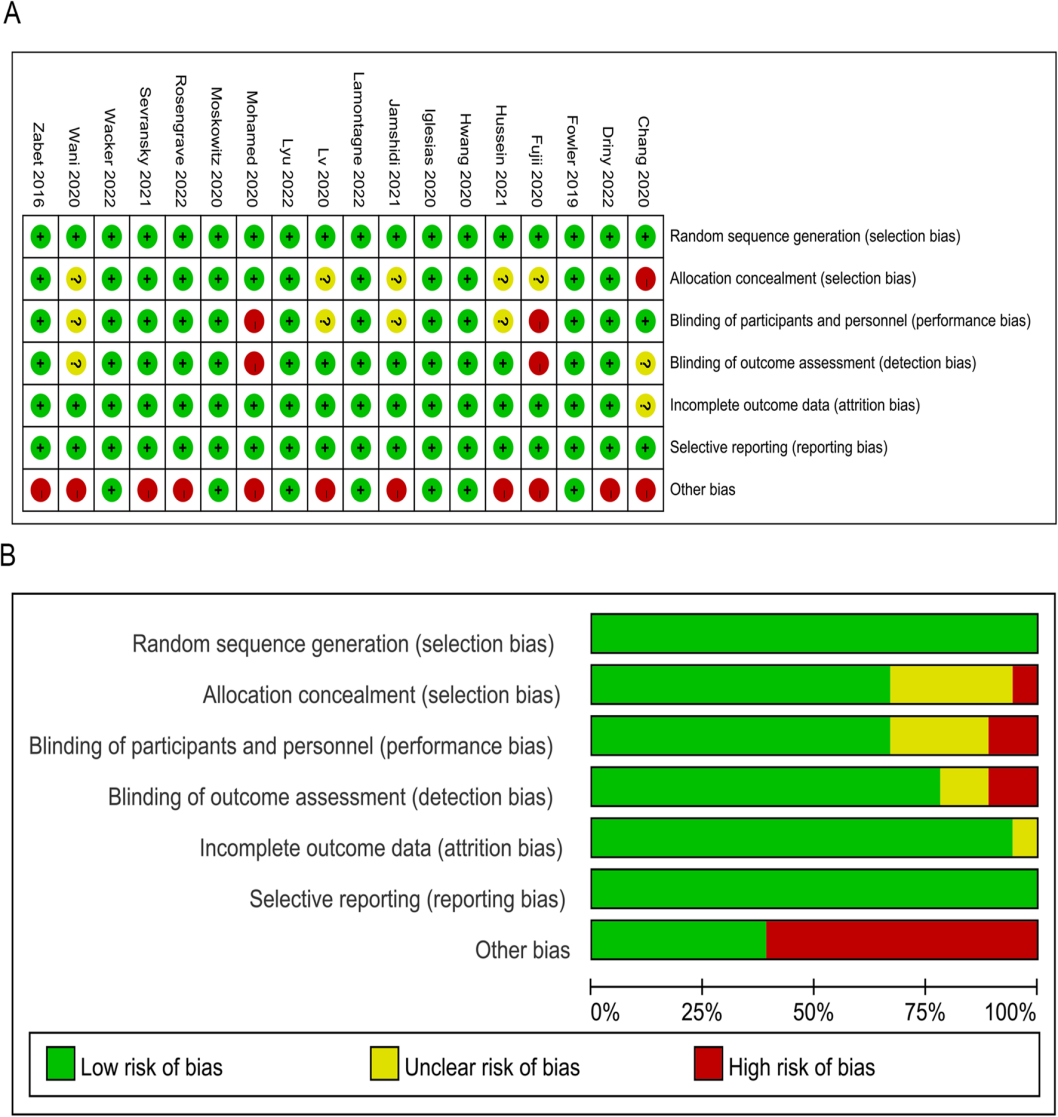
RCTs quality assessment. **A** Risk of bias summary; **B** risk of bias

For random sequence generation, 18 literatures are all low risk of bias; In terms of allocation concealment, 12 articles had low bias risk, 5 article had unclear bias risk, and 1 article had high bias risk; In terms of blinding the subjects and experimenters, 12 literatures are low risk of bias, 2 article had high bias risk and 4 article had unclear bias risk; In terms of blinding the outcome evaluators, 14 literatures are low risk of bias, 2 article had high bias risk, and 2 article had unclear bias risk; In terms of incomplete result data, 17 literatures are low risk of bias, 1 article had unclear bias risk, and 18 literatures are all low risk of bias in selective reporting of research results; For other bias, 7 literatures are low risk of bias and 11 article had high bias risk as shown in Fig. 2.

### Primary outcomes

**Fig. 3:**
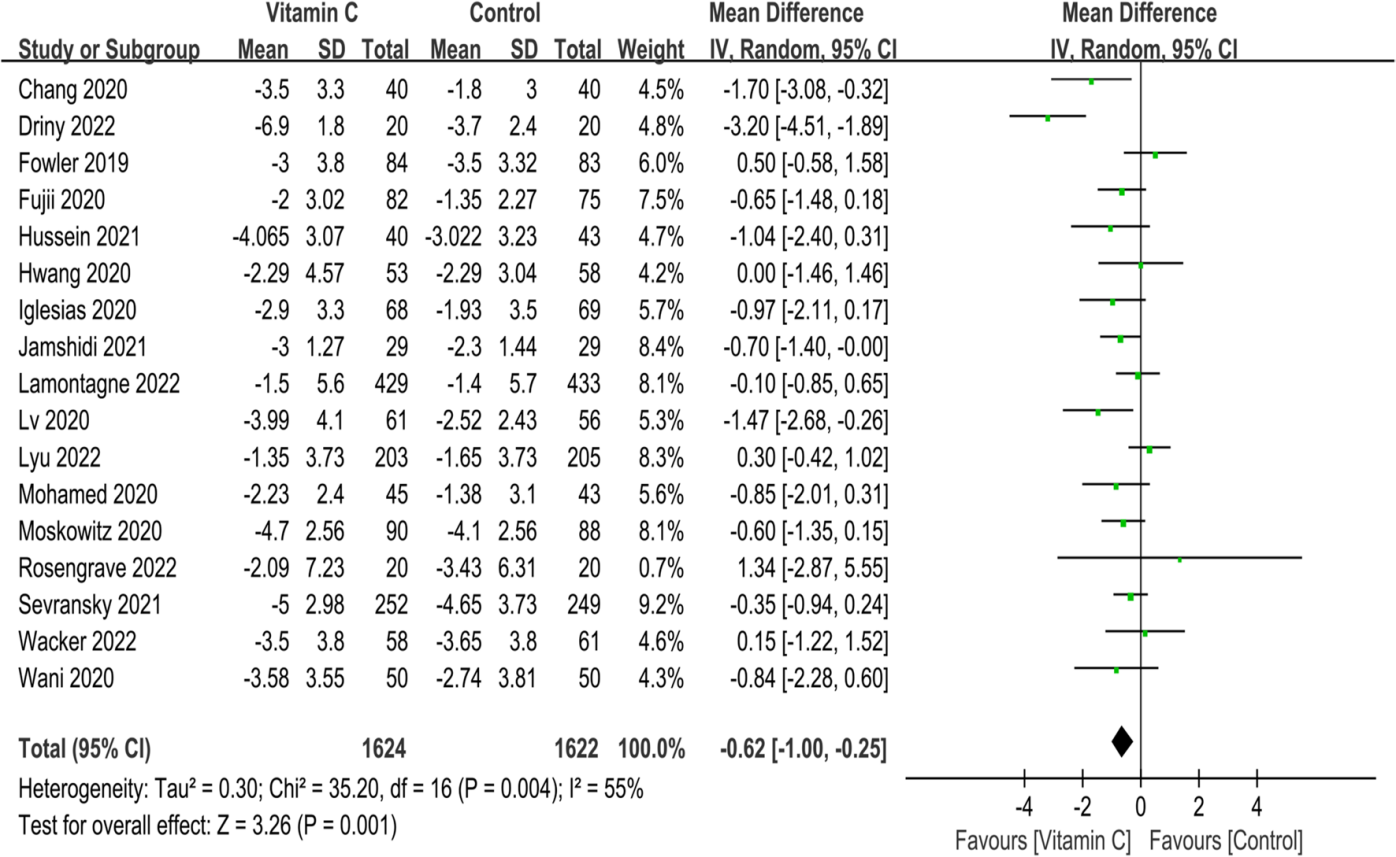
Meta-analysis and forest plot of delta SOFA score

 A total of 17 studies described delta SOFA score [15–17, 24–35, 37, 38], and the use of IVVC was associated with improved delta SOFA score (MD, − 0.62; 95% CI, − 1.00 to − 0.25; *p* = 0.001; *I*^2^ = 55%) (Fig. 3).

### Secondary outcomes

**Fig. 4:**
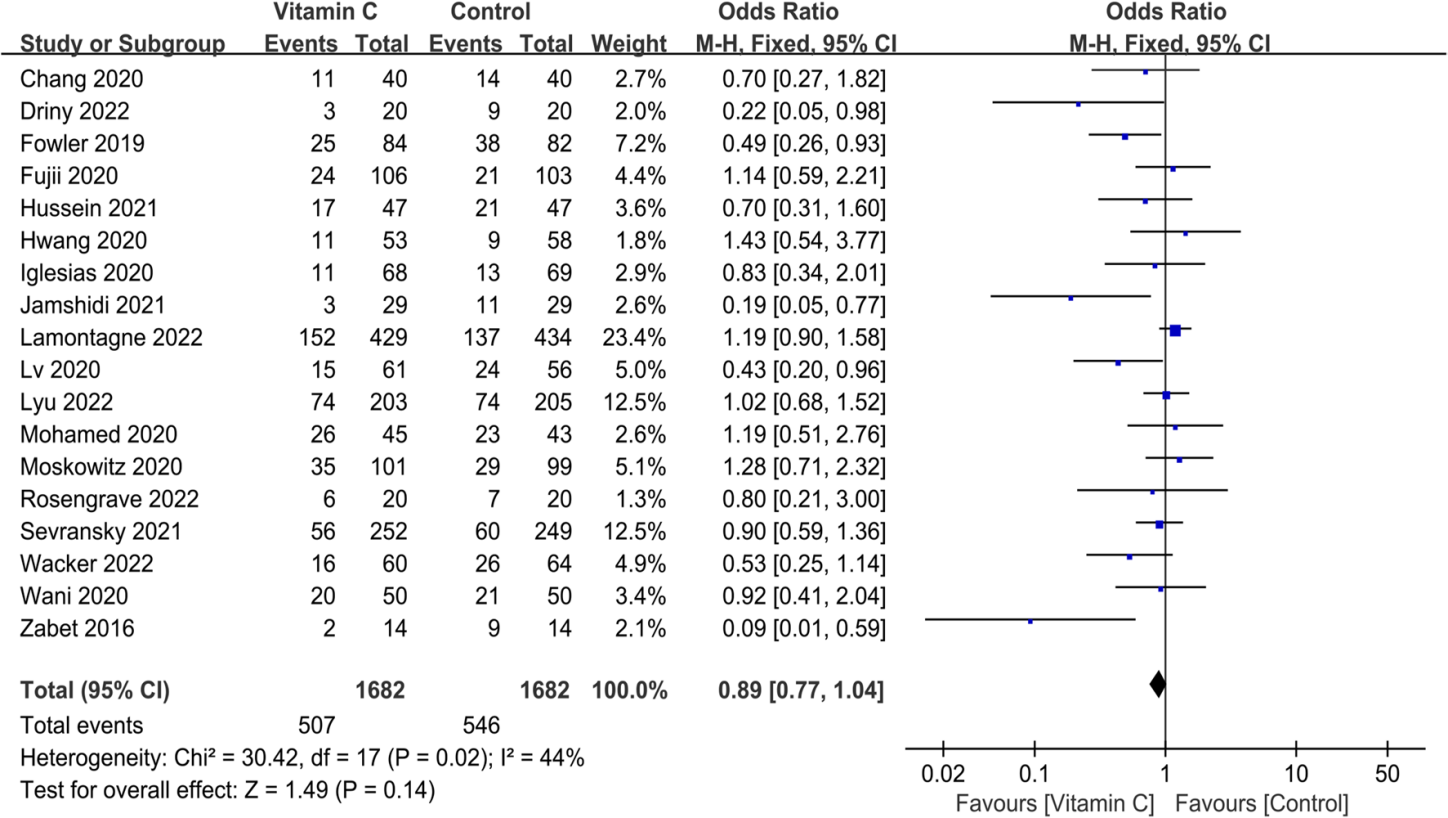
Meta-analysis and forest plot of short-term mortality

**Fig. 5:**
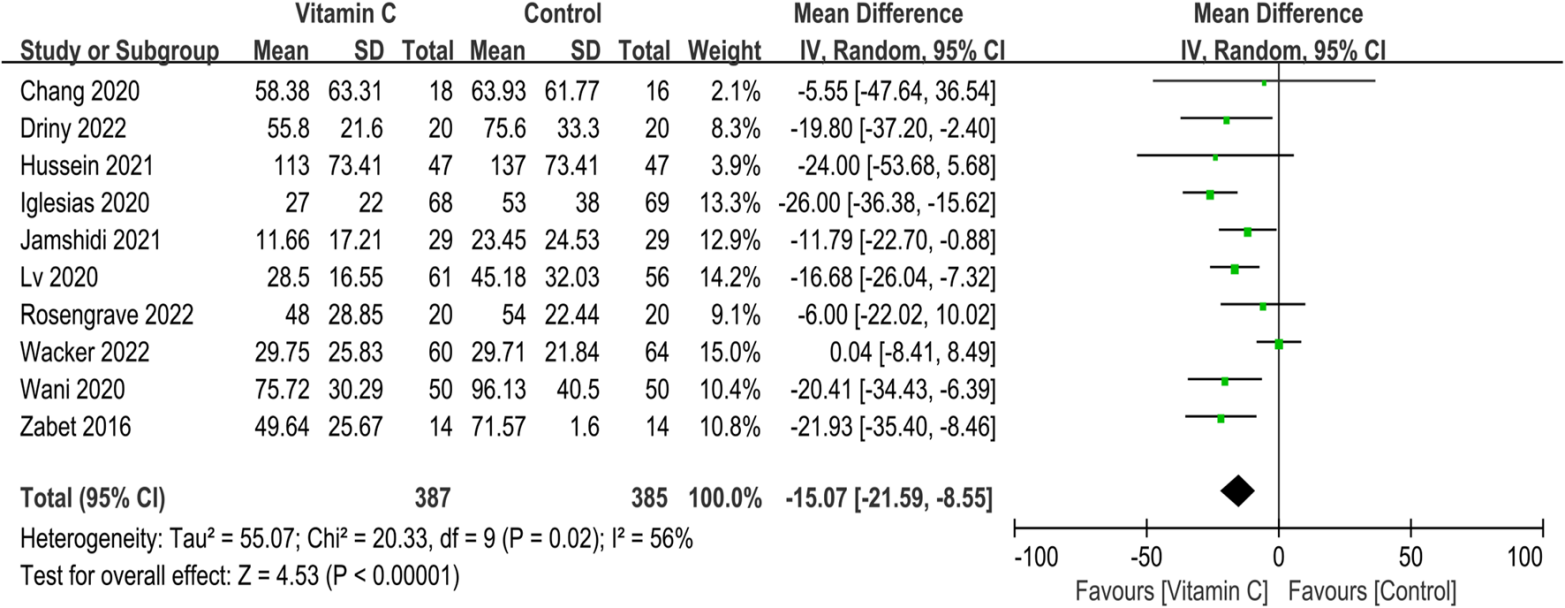
Meta-analysis and forest plot of the duration of vasopressor use

**Fig. 6:**
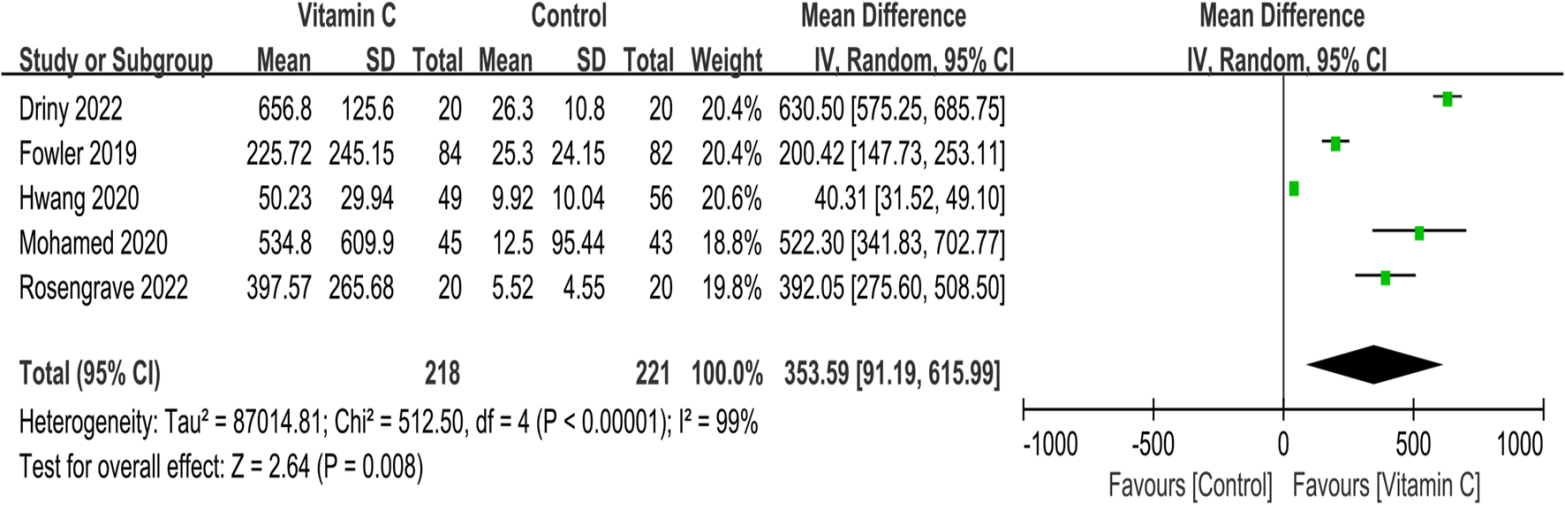
Meta-analysis and forest plot of IV vitamin C level

**Fig. 7:**
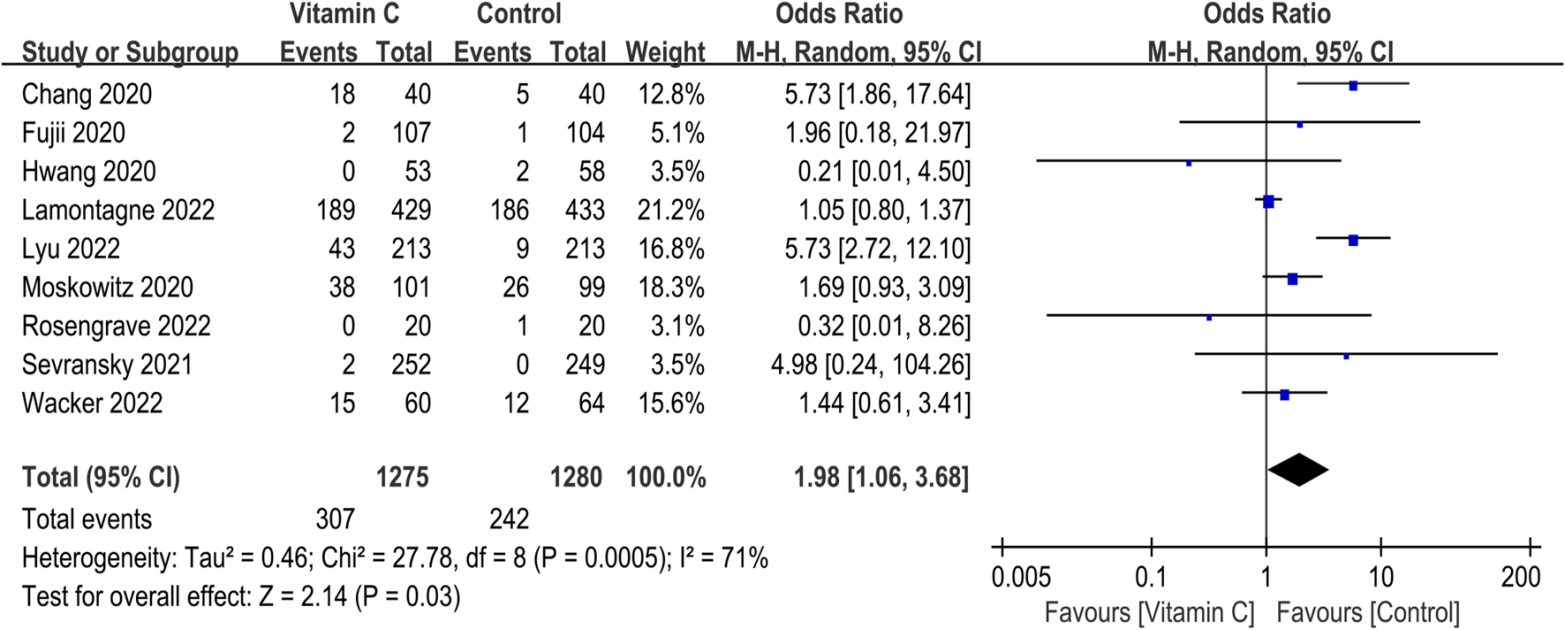
Meta-analysis and forest plot of adverse events

Short-term mortality of all included 18 RCTs is presented in Fig. 4. The pooled result indicated that compared with control group, IVVC was not associated with a statistically significant reduction in short-term mortality (OR, 0.89; 95% CI, 0.77 to 1.04; *p* = 0.14; *I*^2^ = 44%). Ten studies reported the duration of vasopressor use [16, 17, 25–27, 29, 31, 32, 36, 37], and IVVC demonstrated remarkable shorter the duration of vasopressor use (MD, − 15.07; 95% CI, − 21.59 to − 8.55; *p* < 0.00001; *I*^2^ = 56%) (Fig. 5). Five studies reported IVVC level [16, 28, 33, 35, 37], and the vitamin C level was associated with a significantly increase (MD, 353.59; 95% CI, 91.19 to 615.99; *p* = 0.008; *I*^2^ = 99%) (Fig. 6). Night studies reported adverse events [15–17, 24, 28–30, 34, 38] with significant difference between two groups (OR, 1.98; 95% CI, 1.06 to 3.68; *p* = 0.03; *I*^2^ = 71%) (Fig. 7). Notably, substantial heterogeneity was observed in the duration of vasopressor use (*I*^2^ = 56%), vitamin C level (*I*^2^ = 99%) and adverse events (*I*^2^ = 71%).

### Subgroup analysis of delta SOFA score and short-term mortality

#### Dose

No study administered a dose of < 25 mg/kg/d, 14 studies [16, 17, 24–34, 37] (15 studies for short-term mortality [16, 17, 24–34, 36, 37]) administered 25–100 mg/kg/d, and 3 studies [15, 35, 38] administered > 100 mg/kg/d. 25–100 mg/kg/d IVVC administration was associated with reduced delta SOFA score (MD, − 0.85; 95% CI, − 1.23 to − 0.46; *p* < 0.0001; *I*^2^ = 42%) and short-term mortality (OR, 0.80; 95% CI, 0.65 to 0.97; *p* = 0.03; *I*^2^ = 36%) as shown in Table 1.

**Table 1:** Subgroup analysis of delta SOFA score and short-term mortality

Subgroup	Delta SOFA score	Short-term mortality
Overall effect	MD = − 0.62 [− 1.00 to − 0.25], *p* = 0.001	OR = 0.89 [0.77 to 1.04], *p* = 0.14
Dose		
< 25 mg/kg/d	Not estimable	Not estimable
25–100 mg/kg/d	MD = − 0.85 [− 1.23 to − 0.46], *p* < 0.0001	OR = 0.80 [0.65 to 0.97], *p* = 0.03
> 100 mg/kg/d	MD = 0.18 [− 0.29 to 0.65], *p* = 0.45	OR = 1.02 [0.82 to 1.27], *p* = 0.84
Therapy regimens		
Combined therapy	MD = − 0.53 [− 0.81 to − 0.25], *p* = 0.0002	OR = 0.95 [0.78 to 1.16], *p* = 0.64
Monotherapy	MD = − 0.66 [− 1.83 to 0.50], *p* = 0.26	OR = 0.82 [0.66 to 1.03], *p* = 0.09
Duration		
< 96 h	MD = 0.00 [− 1.46 to 1.46], *p* = 1.00	OR = 0.72 [0.33 to 1.58], *p* = 0.41
96 h	MD = − 0.50 [− 0.91 to − 0.08], *p* = 0.02	OR = 1.0 [0.79 to 1.27], *p* = 1.00
Unclear	MD = − 0.76 [− 1.32 to − 0.20], *p* = 0.008	OR = 0.84 [0.69 to 1.02], *p* = 0.08
Patient		
Sepsis	MD = − 1.47 [− 2.68 to − 0.26], *p* = 0.02	OR = 0.43 [0.2 to 0.96], *p* = 0.04
Septic shock	MD = − 0.41 [− 0.73 to − 0.09], *p* = 0.01	OR = 0.9 [0.71 to 1.13], *p* = 0.37
Unclear	MD = − 0.86 [− 1.63 to − 0.08], *p* = 0.03	OR = 0.93 [0.77 to 1.14], *p* = 0.49

#### The type of therapy

Eleven studies [24–31, 33, 34, 38] analyzed IVVC combined with hydrocortisone and thiamine (combined therapy); among them, one study included only vitamin C and thiamine [28]. Seven studies [15–17, 32, 35–37] (6 studies for delta SOFA score [15–17, 32, 35, 37]) tested IVVC alone (monotherapy group). As shown in Table 1, there was prominent effect on delta SOFA score in combined therapy subgroup (MD, − 0.53; 95% CI, − 0.81 to − 0.25; *p* = 0.0002; *I*^2^ = 6%).

#### Duration

Two studies [28, 36] (1 studies for delta SOFA score [28]) analyzed IVVC duration of < 96 h. Five studies [15, 17, 25, 27, 33] tested IVVC duration of 96 h. Eleven studies [16, 24, 26, 29–32, 34, 35, 37, 38] analyzed IVVC of unclear duration. The result suggested superiority of IVVC therapy in alleviating delta SOFA score at 96 h (MD, − 0.50; 95% CI, − 0.91 to − 0.08; *p* = 0.02; *I*^2^ = 0%) and unclear (MD, − 0.76; 95% CI, − 1.32 to − 0.20; *p* = 0.008; *I*^2^ = 68%) subgroup (Table 1).

#### Patient

One study [32] analyzed sepsis patients. Ten studies [16, 17, 25, 26, 28, 30, 33, 34, 36, 38] tested septic shock patient (9 studies for delta SOFA score [16, 17, 25, 26, 28, 30, 33, 34, 38]). Seven studies [15, 24, 27, 29, 31, 35, 37] analyzed unclear patients. As illustrated in Table 1, there were remarkable effects on delta SOFA score (MD, − 1.47; 95% CI, − 2.68 to − 0.26; *p* = 0.02) and short-term mortality (OR, 0.43; 95% CI, 0.20 to 0.96; *p* = 0.04) in the sepsis patients but only with one study.

### Publication bias and sensitivity analysis

**Fig. 8:**
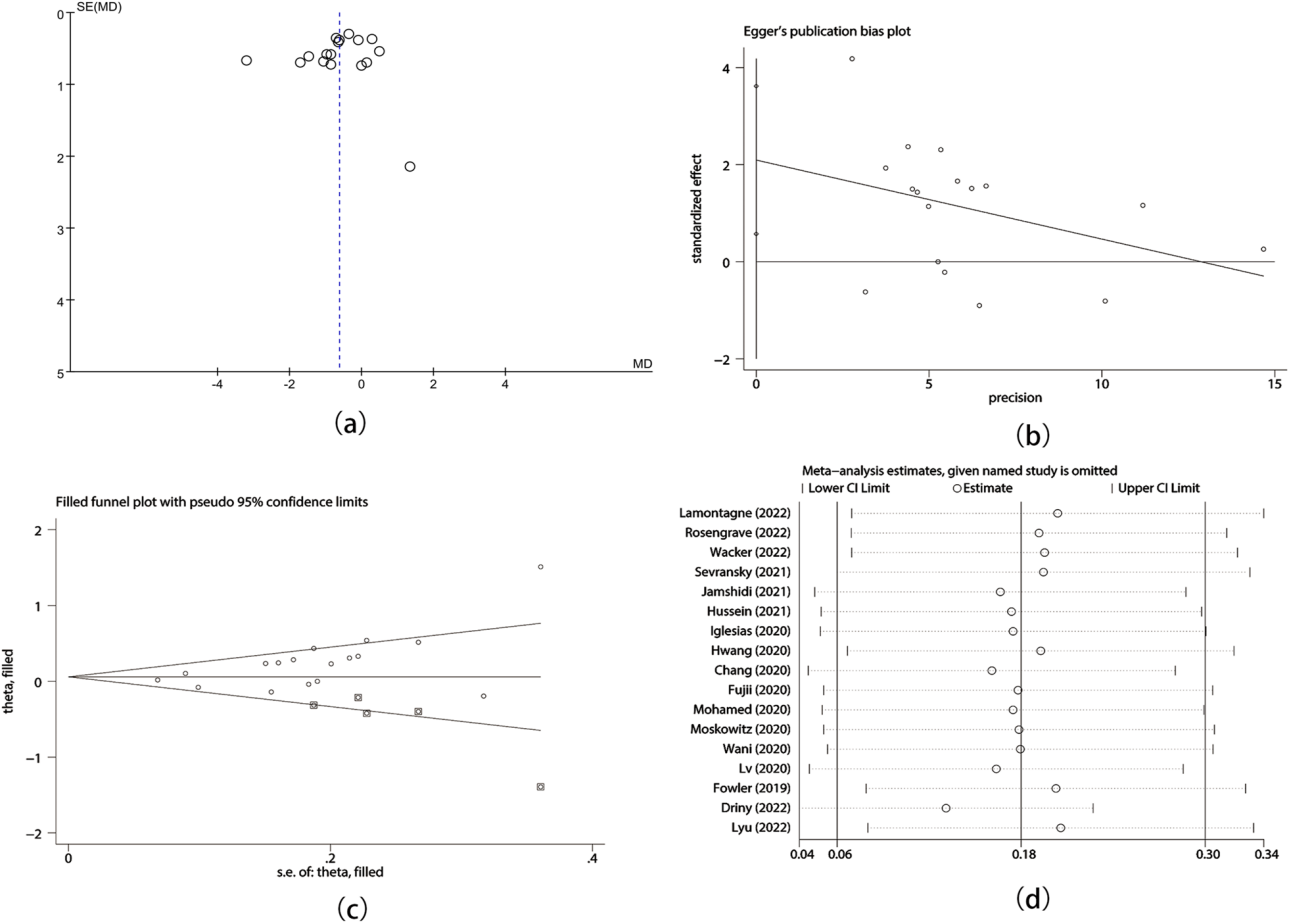
Publication bias and sensitivity analysis of delta SOFA score. (**a**) Funnel plot; (**b**) Egger’s test; (**c**) Trim and fill analysis; (**d**) Sensitivity analysis

**Fig. 9:**
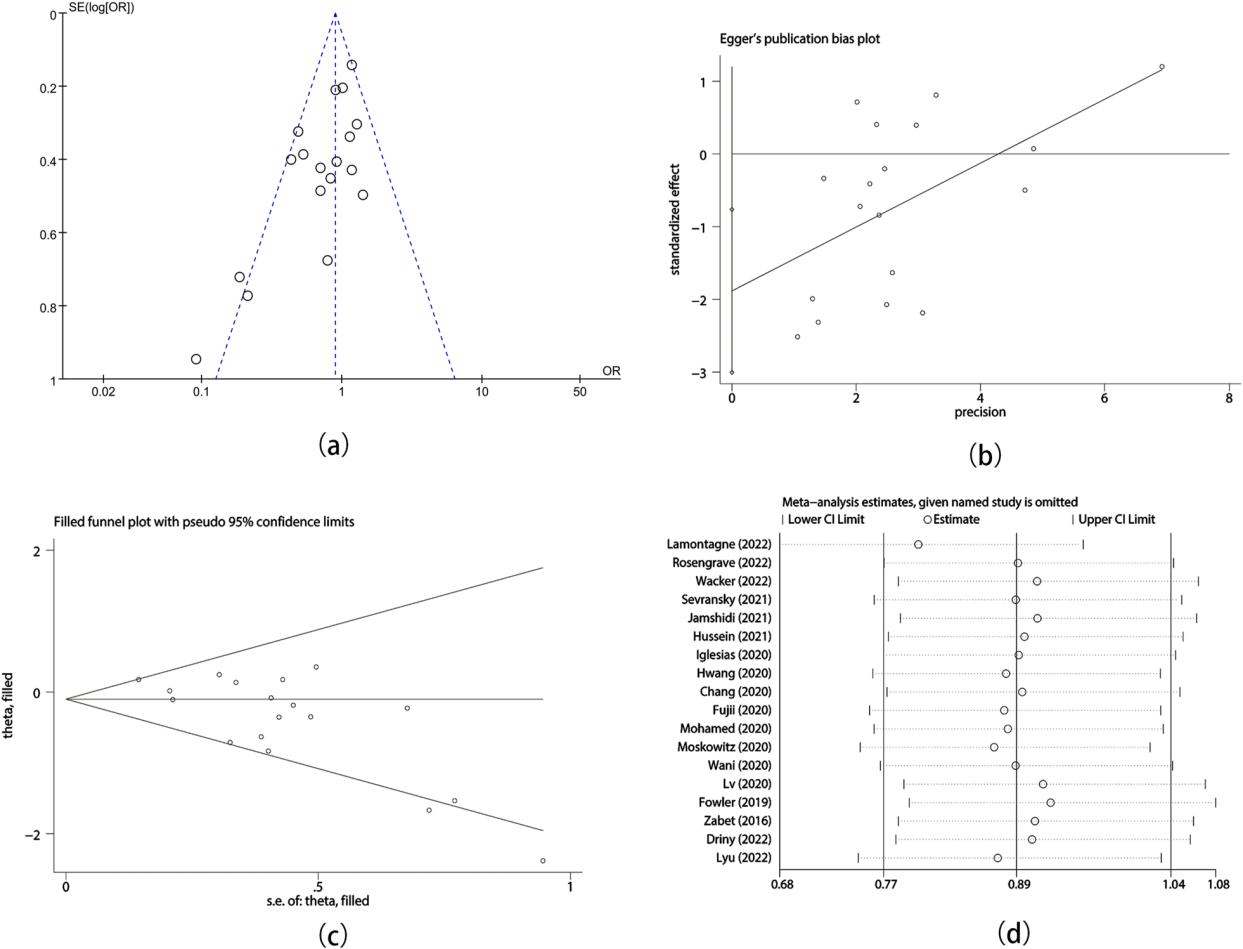
Publication bias and sensitivity analysis of short-term mortality. (**a**) Funnel plot; (**b**) Egger’s test; (**c**) Trim and fill analysis; (**d**) Sensitivity analysis

Funnel plots were conducted to evaluate for publication bias for delta SOFA score and short-term mortality, and we detected asymmetry when visually assessing. We also statistically evaluated publication bias through Egger’s test. The result revealed that there was publication bias in the RCTs on delta SOFA score (*p* = 0.01) and short-term mortality (*p* = 0.003). The meta-analysis result for mortality was robust in further trim and fill analysis. To find out the source of heterogeneity, we used sensitivity analysis for included RCTs and suggested that Lamontagne’s study was the source of heterogeneity in short-term mortality (Figs. 8a–d and 9a–d).

### Meta-regressions analysis

Meta-regressions analysis of delta SOFA score and short-term mortality was performed to find out the specific influencing factors of heterogeneity. The results of meta-regressions analysis showed that different type of therapy (*p* = 0.02) was significantly affected short-term mortality factor (Additional file 1: Fig. S1). There was no subgroup significantly affecting delta SOFA score (Additional file 1: Fig. S2).

### Certainty of the evidence

There was a statistically difference on delta SOFA score with IVVC administration, which was categorized as moderate-quality evidence. IVVC administration indicated no statistically significant effect on short-term mortality with moderate-quality based on GRADE criteria. Compared to control group, treatments containing vitamin C were associated with significant shorter vasopressor duration. The GRADE summary of primary outcome and secondary outcomes is presented in Tables 2 and 3.

**Table 2:** GRADE summary of results for the primary outcome

Outcomes	No. of studies	Sample size		Absolute effect estimates (95% CI)	Certainty (GRADE)
		Vitamin C	Control		
Delta SOFA score	17	1624	1622	− 0.62 (− 1.00 to − 0.25)	Moderate
Dose—< 25 mg/kg/d	0	0	0	–	–
Dose—25–100 mg/kg/d	14	908	901	− 0.85 (− 1.23 to − 0.46)	Moderate
Dose—> 100 mg/kg/d	3	716	721	0.18 (− 0.29 to 0.65)	Moderate
Therapy regimens—combined therapy	11	952	949	− 0.53 (− 0.81 to − 0.25)	Low
Therapy regimens—monotherapy	6	672	673	− 0.66 (− 1.83 to 0.50)	Low
Duration—< 96 h	1	53	58	0 (− 1.46 to 1.46)	Moderate
Duration—96 h	5	629	635	− 0.50 (− 0.91 to − 0.08)	High
Duration—unclear	11	942	929	− 0.76 (− 1.32 to − 0.20)	Very low
Patient—sepsis	1	61	56	− 1.47 (− 2.68 to − 0.26)	Low
Patient—septic shock	9	620	622	− 0.41 (− 0.73 to − 0.09)	Low
Patient—unclear	7	943	944	− 0.86 (− 1.63 to − 0.08)	Very low

**Table 3:** GRADE summary of results for secondary outcomes

Outcomes	Odds ratio (95% CI)	Sample size		No. of studies (total patients)	Certainty (GRADE)
		Vitamin C	Control		
Short-term mortality	0.89 (0.77 to 1.04)	507/1682	546/1682	18 (3364)	Moderate
Dose—< 25 mg/kg/d	–	–	–	0 (0)	–
Dose—25–100 mg/kg/d	0.80 (0.65 to 0.97)	256/966	297/961	15 (1927)	High
Dose—> 100 mg/kg/d	1.02 (0.82 to 1.27)	251/716	249/721	3 (1437)	Low
Therapy regimens—combined therapy	0.95 (0.78 to 1.16)	288/994	296/992	11 (1986)	Moderate
Therapy regimens—monotherapy	0.82 (0.66 to 1.03)	219/688	250/690	7 (1378)	Low
Duration—< 96 h	0.72 (0.33 to 1.58)	13/67	18/72	2 (139)	Very low
Duration—96 h	1 (0.79 to 1.27)	208/631	210/639	5 (1270)	Low
Duration—Unclear	0.84 (0.69 to 1.02)	286/984	318/971	11 (1955)	Moderate
Patient—sepsis	0.43 (0.2 to 0.96)	15/61	24/56	1 (117)	Moderate
Patient—septic shock	0.9 (0.71 to 1.13)	214/678	230/682	10 (1360)	Moderate
Patient—unclear	0.93 (0.77 to 1.14)	278/943	292/944	7 (1887)	Moderate
Duration of vasopressor use	− 15.07 (− 21.59 to − 8.55)	387	385	10 (772)	Moderate
IV vitamin C level	353.59 (91.19 to 615.99)	218	221	5 (439)	Low
Adverse events	1.98 (1.06 to 3.68)	307/1275	242/1280	9 (2555)	Moderate

### Trial sequential analysis

**Fig.10:**
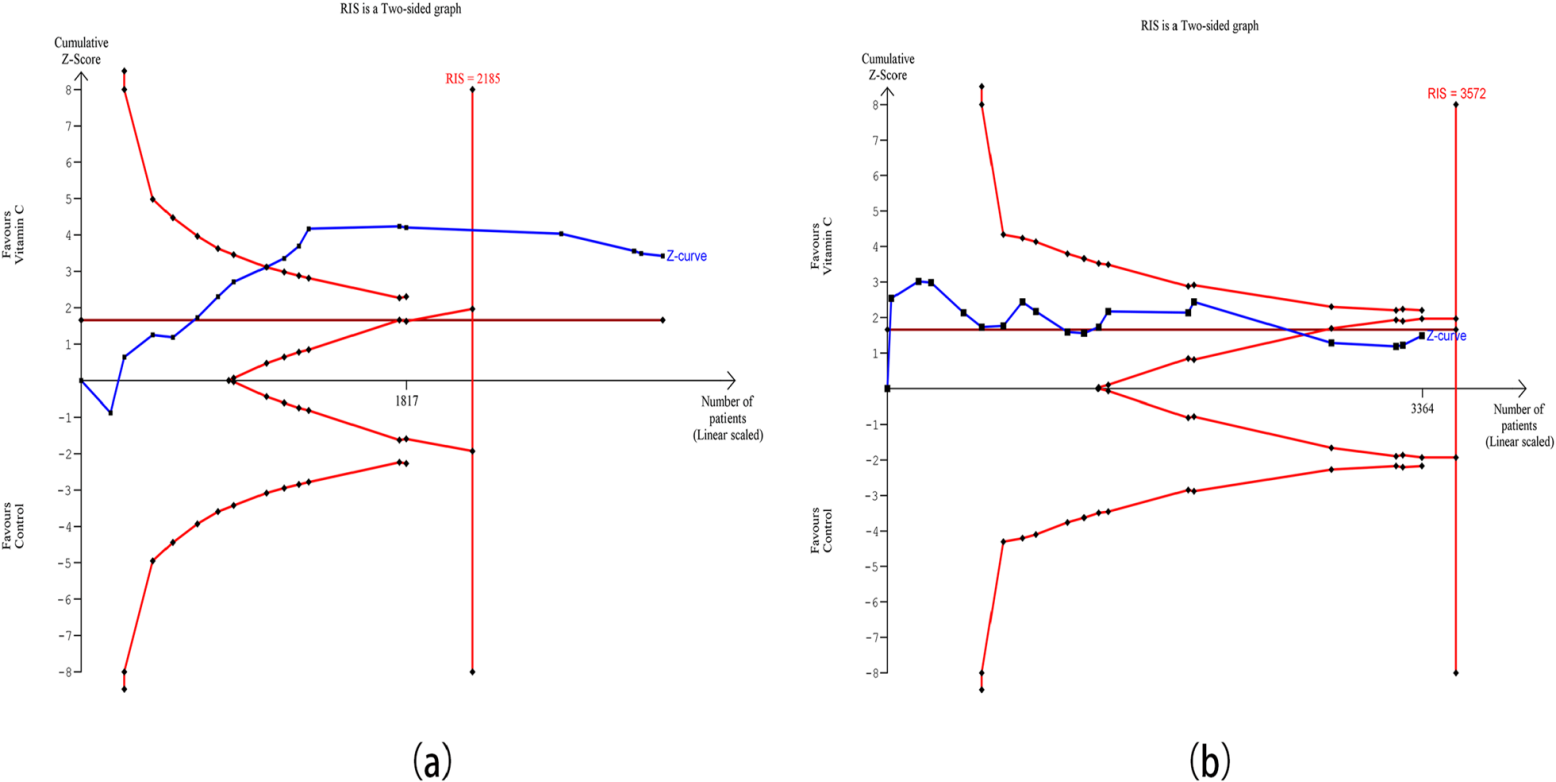
Trial sequential analysis for delta SOFA score (**a**) and short-term mortality (**b**)

Uppermost and lowermost curves represent trial sequential monitoring boundary lines for benefit and harm, respectively. Horizontal lines represent the traditional boundaries for statistical significance. Triangular lines represent the futility boundary. The cumulative Z curve represents the trial data. As shown in Fig. 10a, the TSA showed that the RIS of delta SOFA score has been achieved. The cumulative Z curve crossed both the traditional boundary and the trial sequential monitoring boundary, hinting that it was true positive result for delta SOFA score. By performing TSA, the RIS was calculated to be 3572 patients for short-term mortality, and the cumulative Z curve did not exceeded the RIS. With the increase in RCTs, the cumulative Z curve neither crossed the traditional boundary nor crossed the trial sequential monitoring boundary, but crossed the futility boundary, indicating that currently cumulative evidence is infinitely close to the true value with a negative result (Fig. 10b).

## Discussion

We carried out this meta-analysis of the most recent RCTs to analyze the efficacy of IVVC in sepsis or septic shock patients. Eighteen RCTs with 3364 patients were included in the final analysis. To our knowledge, this is the first comprehensive meta-analysis not only added the latest RCTs that were published recently, and some were not included in previous meta-analysis, but also applied sensitivity analysis, subgroup analysis, regression analysis, GRADE and TSA to estimate and confirm the effects of IVVC in sepsis or septic shock patients.

Based on sepsis 3.0, SOFA score is an important tool to evaluate the organ function and critical degree of septic patients. It was reported that therapeutic efficacy on delta SOFA score seems to be reliably and consistently associated with mortality in RCTs [39]. An increase in 2 points or more of SOFA score (from baseline) was related to an approximate 10% increase in mortality [40]. Delta SOFA score would suggest whether organ function has improved, and has been chosen as primary outcome in several RCTs involving patients with sepsis or septic shock, together with reporting mortality [27, 28, 34, 35, 37]. Additionally, given that mortality is influenced by many factors, the delta SOFA score was chosen as the primary outcome in our meta-analysis. Although several RCTs [27, 28, 34, 35] which delta SOFA score as primary outcome found that IVVC did not significantly improve organ function compared with placebo, our meta-analysis indicated promising results that IVVC could significantly improve delta SOFA score after integrating multiple RCTs, which was validated by TSA. Thus, it is unlikely that further trials will change the conclusion and are not necessary. It is worth noting that there was medium degree of heterogeneity in the pool results of delta SOFA score, and publication bias was found in the assessment of Egger’s test. However, there was no subgroup significantly affecting delta SOFA score in meta-regressions analysis.

The outcome analysis revealed IVVC in sepsis or septic shock patients was not associated with improved short-term mortality. In addition, the conclusion could be reached without incorporating additional RCTs based on the result of TSA. There was no statistically difference between IVVC combination therapy and monotherapy. The outcome was contrary to the study of Marik et al. [8], which suggested that combined treatment could decrease hospital mortality.

Triple therapy of vitamin C, hydrocortisone and thiamine has biological rationality in sepsis; however, our outcome was insignificant in contrast with studies which concluded that coadministration offered acceptable outcomes in sepsis or septic shock patients [8, 25]. On the other hand, several large RCTs of vitamin C in sepsis or septic shock did not produce a remarkable reduction in mortality [15, 24, 30, 34]. Despite the large multicenter trial of VITAMINS, there was a concern about the time of combined therapy initiation [30]. Individual trial of combination treatment had shown a decrease in 28-day mortality in the prespecified subgroup of sepsis patients within 48 h speculate that administering vitamin C as soon as possible may be meaningful [29]. However, this result has not been replicated. As sepsis is a very time-sensitive disease, earlier intervention is associated with better outcomes. It is possible that a shorter time from ICU admission or vasopressor initiation to intervention may have improved outcomes. However, there are only 9 RCTs presenting time to intervention initiation in our meta-analysis, resulting in difficulty in judgement the effect of initiation time. Recent meta-analyses [11, 13, 41–43] also failed to find improved mortality among patients with sepsis, no matter combined therapy or monotherapy. Different inclusion criterion, duration, patient populations and timing of administration explain the heterogeneity of different research results to some extent.

We attempted to determine whether there may be some significant differences between high and low dose of vitamin C by performing a subgroup analysis comparing mortality. Interestingly, our results revealed that dose at 25–100 mg/kg/d IVVC was associated with improved short-term mortality, which was contrary to previous meta-analysis that high-dose (≥ 10 g/d) IVVC was benefit for mortality. CITRIS-ALI trial revealed that compared with placebo, high-dose vitamin C (50 mg/kg every 6 h) had a lower 28-day mortality in exploratory analysis [35], while the larger-scale LOVIT trial did not improve 28-day mortality [15]. In addition, a component network meta-analysis demonstrated high-dose (> 6 g/d) and very-high dose vitamin C (> 12 g/d) was associated with decreased mortality but with low certainty [44]. The exact mechanism by which low-dose vitamin C works has not been clarified. However, these studies populations of previous meta-analysis included critically ill patients. It is unclear whether higher doses confer greater benefits; although low dose was used in VITAMINS trial [30], the vitamin C level reached almost the same concentration at 6 h [45] as published in CITRIS-ALI trial [35] of a high dosing regimen at 48 h.

In spite of mortality, this meta-analysis indicated promising results that IVVC could significantly shorten the duration of vasopressor use. Our results were contrary to meta-analysis of Cai et al. [11] that vitamin C did not produce obvious effect on the duration of vasopressor use; however, this meta-analysis comprised 6 cohort studies. On the other hand, our results were in line with recent meta-analyses which only included RCTs [13, 46]. Corticosteroids decrease vasopressor requirements in septic shock [47, 48], and the hemodynamic improvement may be due to corticosteroids [34]. Vitamin C has numerous effects including antioxidant, immune-supporting, anti-inflammatory and improving vascular endothelial cell function [6, 49]. As is well known, vitamin C is a cofactor in the production of biosynthetic enzymes that can increase vasopressin synthesis [6, 50]. Therefore, the vital role of vitamin C may be the reason that vitamin C therapy could reduce the duration of vasopressor use and improve delta SOFA score. Hypovitaminosis is generally recognized as vitamin C level below 23 μmol/L [27]. A previous study showed that patients with sepsis need 3 g/day of vitamin C to reach normal plasma level [36]. Five trials enrolled in our meta-analysis showed the vitamin C level in intervention group at 72–96 h was increased significantly, but did not translate into improved clinical mortality. A previous study suggested that some patients may develop vitamin C deficiency within 48 h after discontinuing vitamin C infusion in spite of the dosing regimen [51], which may result in no improvement in mortality.

Adverse effects of vitamin C were rare. Driny et al. believed that vitamin C was safe, tolerable and would not cause patients withdraw from the study [37]. On the basis of this study, Fowler et al. reported that different doses of vitamin C would not lead to any serious adverse events [7]. No studies have shown that IVVC significantly increases adverse events except for Chang’s and Lyu’s studies. Chang’s study was discontinued due to the high incidence of severe hypernatremia after interim analysis, which was attributed to the adverse effects of hydrocortisone [29]. Our meta-analysis demonstrated higher adverse events in the vitamin C group; however, this result should be interpreted with caution due to medium heterogeneity.

We acknowledge some limitations. First of all, 10 RCTs [16, 25, 26, 29, 31–33, 36–38] were all single-center studies, which may result in selective bias, so as to obtain a large beneficial therapeutic effect conclusion. Second, RCTs in this meta-analysis used different doses of vitamin C. Most RCTs administered 1.5 g vitamin C every 6 h or 25 mg/kg every 6 h, whereas other RCTs administered 50 mg/kg every 6 h or 200 mg/kg daily. We consider that the difference dose of vitamin C was likely to affect the efficacy of the meta-analysis, but our meta-regressions analysis indicated that dose was not associated with outcomes of delta SOFA score and short-term mortality. Third, the duration of vitamin C may have obvious impact on the outcome. Many patients in the intervention group did not administrate the entire duration of IVVC [16, 34], which may reduce benefit of the treatment group to some extent. Moreover, some trials [24, 29] early terminated their RCTs for reasons, which may also be one of the reasons for different outcomes.

## Conclusion

To our knowledge, this is the most comprehensive meta-analysis including newer and the largest RCT of Famontagne et al. to update of vitamin C treatment in sepsis or septic shock. In this meta-analysis, IVVC in sepsis or septic shock patients significantly improved delta SOFA score and reduced the duration of vasopressor use, whereas it was not associated with reduction in short-term mortality and had higher adverse events.

## Supplementary Information

**Additional file 1.** **Table S1**. Search Strategy. **Table S2**. Baseline Characteristics of Included RCTs. **Fig. S1**. Meta-regressions analysis of delta SOFA score. **Fig. S2**. Meta-regressions analysis of short-term mortality.

## Data Availability

The datasets used and/or analyzed during the current study are available from the corresponding author on reasonable request.

## Abbreviations

RCTs: Randomized controlled trials
IVVC: IV vitamin C
SOFA: Sequential Organ Failure Assessment
OR: Odds ratio
MD: Mean difference
CIs: Confidence intervals
IQRs: Interquartile ranges
SD: Standard deviation
GRADE: Grading of Recommendations, Assessment, Development and Evaluations
TSA: Trial sequential analysis
RIS: Required information size
