# Supplementary material for: The outcome of IV vitamin C therapy in patients with sepsis or septic shock: a meta-analysis of randomized controlled trials
Source: Crit Care. 2023 Mar 13;27:109. doi: 10.1186/s13054-023-04392-y (PMC10012592; doi:10.1186/s13054-023-04392-y)
Supplement: Supplementary file 1 — Additional file 1. Table S1. Search Strategy. Table S2. Baseline Characteristics of Included RCTs. Fig. S1. Meta-regressions analysis of delta SOFA score. Fig. S2. Meta-regressions analysis of short-term mortality. [file 13054_2023_4392_MOESM1_ESM.docx]

**The Outcome of IV Vitamin C Therapy in Patients with Sepsis or Septic Shock: A Meta-Analysis of Randomized Controlled Trials**

**Supplemental content for The Outcome of IV Vitamin C Therapy in Patients with Sepsis or Septic Shock: A Meta-Analysis of Randomized Controlled Trials**

**Table S1.** Search Strategy.

**Table S2.** Baseline Characteristics of Included RCTs.

**Fig. S1.** Meta-regressions analysis of delta SOFA score.

**Fig. S2.** Meta-regressions analysis of short-term mortality.

**Table S1.** Search Strategy.

| Database | Date of search | Search items | Results found |
| --- | --- | --- | --- |
| PubMed | June 20th, 2022 | #1: "Ascorbic Acid"[MeSH Terms]  #2: "ascorbic"[Title/Abstract] OR "ascorbate"[Title/Abstract] OR "vitamin c"[Title/Abstract] OR "antioxidant*"[Title/Abstract]  #3: "Sepsis"[MeSH Terms]  #4: "Sepsis"[Title/Abstract] OR "septic shock"[Title/Abstract]  #5: "randomized controlled trial"[Title/Abstract] OR "controlled clinical trial"[Title/Abstract] OR "randomized controlled trials"[Title/Abstract] OR "blind*"[Title/Abstract] OR "clinical trial*"[Title/Abstract] OR "placebo*"[Title/Abstract] OR "random*"[Title/Abstract]  #6: "Ascorbic Acid"[MeSH Terms] OR "ascorbic"[Title/Abstract] OR "ascorbate"[Title/Abstract] OR "vitamin c"[Title/Abstract] OR "antioxidant*"[Title/Abstract]  #7: "Sepsis"[MeSH Terms] OR "Sepsis"[Title/Abstract] OR "septic shock"[Title/Abstract]  #8: ("Ascorbic Acid"[MeSH Terms] OR ("ascorbic"[Title/Abstract] OR "ascorbate"[Title/Abstract] OR "vitamin c"[Title/Abstract] OR "antioxidant*"[Title/Abstract])) AND ("Sepsis"[MeSH Terms] OR ("Sepsis"[Title/Abstract] OR "septic shock"[Title/Abstract])) AND ("randomized controlled trial"[Title/Abstract] OR "controlled clinical trial"[Title/Abstract] OR "randomized controlled trials"[Title/Abstract] OR "blind*"[Title/Abstract] OR "clinical trial*"[Title/Abstract] OR "placebo*"[Title/Abstract] OR "random*"[Title/Abstract]) | 396 |
| Embase | June 20th, 2022 | #1: 'ascorbic acid'/exp  #2: 'ascorbic acid'  #3: 'ascorbic'  #4: 'ascorbate'  #5: 'vitamin c'  #6: 'antioxidant'  #7: 'sepsis'/exp  #8: 'sepsis'  #9: 'septic shock'  #10: 'randomized controlled trial'  #11: 'controlled clinical trial'  #12: 'randomized controlled trials'  #13: #1 OR #2 OR #3 OR #4 OR #5 OR #6  #14: #7 OR #8 OR #9  #15: #10 OR #11 OR #12  #16: #13 AND #14 AND #15 | 398 |
| Clinical Trials.gov | June 20th, 2022 | #1: Condition or disease:Sepsis  #2: Study Results: Studies With Result  #3:Intervention/treatment: Vitamin C | 9 |

**Table S2.** Baseline Characteristics of Included RCTs.

| References | Research Institute | Classification of Diseases | Type of  Therapy | Time to   therapy  initiation | Glucocorticoid use | Patient, n | Age | Male,  n ( %) | SOFA Score | Vasopressors, n ( %) | Mechanical  Ventilation, n ( %) |
| --- | --- | --- | --- | --- | --- | --- | --- | --- | --- | --- | --- |
| Lamontagne  et al  (LOVIT trial)  (15) | International multi-centered clinical trial | Sepsis requiring  vasopressor | Vitamin C  (50 mg/kg q6h for up to 96 h) | Within 4 h after randomization | Administration of glucocorticoids were performed at the discretion of the treating teams (46.4% in vitamin c group and 45.4% in placebo group) | 429 | 65.0±14.0 | 278 (64.8) | 10.2±3.4 | 428 (99.8) | 294 (68.5) |
|  |  |  | Placebo |  |  | 433 | 65.2±13.8 | 260（60.0） | 10.1±3.7 | 433 (100) | 283 (65.4) |
| Rosengrave  et al (16) | New Zealand single center clinical trial | Septic shock | Vitamin C (25 mg/kg q6h, 96 h or until death or discharge) | Not reported | Not reported | 20 | 69 (64-76) | 16 (80) | 8.5 (6.8-11) | 20（100） | Not reported |
|  |  |  | Placebo |  |  | 20 | 66 (57-71) | 11 (55) | 9.0 (7.8-10) | 20（100） | Not reported |
| Wacker  et al (17) | America multi-centered clinical trial | Septic Shock | Vitamin C (1,000 mg bolus over 30min followed by continuous infusion of 250 mg/hr for 96 h) | Not reported | 50% received steroids  in vitamin c group and 65.6% in placebo group | 60 | 68.9  (60.1–79.9) | 30 (50) | 10 (7–11) | 60（100） | 34 (56.7) |
|  |  |  | Placebo |  |  | 64 | 73.0 (60.8–80.0) | 33 (52) | 9 (7–12) | 64（100） | 37 (57.8) |
| Driny  et al (37) | Egypt single center clinical trial | Sepsis  requiring mechanical ventilation | Vitamin C (1.5 g/6 h for 4 consecutive days). Study drug infusion was stopped when the last dose (Dose 16) was administered or at ICU discharge, study withdrawal, or death, which of which happened first | Within 2 hours  after the randomization | Not reported | 20 | 53.0±23.3 | 9 (45) | 12.1± 1.6 | Not reported | 20 (100) |
|  |  |  | 100 mg vitamin C/day as a first single dose and the other three subsequent doses for 4 consecutive days |  |  | 20 | 52.1±18.8 | 12 (60) | 12.7± 2.1 | Not reported | 20 (100) |
| Sevransky  et al  (VICTAS trial)  (24) | America multi-centered clinical trial | Sepsis-induced  respiratory and/or cardiovascular  dysfunction | Vitamin C 1.5 g+thiamine 100 mg+hydrocortisone 50 mg q6h for 96 h or until discharge from the intensive care unit or death | Within 4 hours of randomization | 33% patients in the intervention group and 32% of control patients received clinicianprescribed corticosteroids | 252 | 62 (51-69) | 139 (55.2) | 9 (7-12) | 93 (36.9) | 49 (19.4) |
|  |  |  | Placebo |  |  | 249 | 61 (50-72) | 134 (53.8) | 9 (6-11) | 97/248 (39.1) | 54/248 (21.8) |
| Lyu et al (38) | China single-center trial | Septic shock | Hydrocortisone 200 mg daily, vitamin C 2 g every 6 h, and thiamine 200 mg every 12 h for 5 days or until ICU discharge, whichever  occurred first | Time from randomization to first study drug were 1.0 (0.0–3.0) h for intervention group and 1.0 (1.0–3.0) h for placebo group | Open‑label hydrocortisone: Additional steroids (5:4 for intervention group and 4:9 for placebo group) | 203 | 69.0（60.0-78.0） | 131（64.5） | 10（7-12） | 203 (100) | 168（82.8） |
|  |  |  | Placebo |  |  | 205 | 71.0（62.0-78.5） | 142（69.3） | 9（7-11） | 205 (100) | 179（87.3） |
| Jamshidi  et al (25) | Iran single center clinical trial | Septic shock | Hydrocortisone (50 mg/6h)+Vitamin C (1.5 g/6h ), and thiamine (200 mg/12h ) for up to 4 days | Not reported | Not reported | 29 | 45.4±19.8 | 21 (72.4) | 4.5±1.4 | 29 (100) | Not reported |
|  |  |  | Placebo |  |  | 29 | 45.4±15.8 | 26 (89.7) | 5.3±1.6 | 29 (100) | Not reported |
| Hussein  et al (26) | Egypt single center clinical trial | Septic shock | Hydrocortisone (50 mg/6h for seven-day or ICU discharge followed by tapering)+vitamin C (1.5 g/6h for 4-day or till ICU-discharge)+thiamine (200 mg/12h for 4-day or till ICU-discharge) | Not reported | Not reported | 47 | 65.81 ± 17.02 | 25 (53.2) | 8.79±2.3 | 47 (100) | Not reported |
|  |  |  | Hydrocortisone ( 50 mg/6h for seven-day or till ICU-discharge, if sooner, followed by tapering) |  |  | 47 | 61.60 ± 18.22 | 26 (55.3) | 8.72±2.08 | 47 (100) | Not reported |
| Iglesias  et al  (ORANGES trial) (27) | America multi-centered clinical trial | Septic and septic shock | Ascorbic acid (1,500 mg q6h)+thiamine (200 mg q12h)+hydrocortisone (50 mg q6h for a maximum of 4 days) | Not reported | Not reported | 68 | 70.0 ±12.0 | 32 (47) | 8.3±3 | 56 (82) | 34 (50) |
|  |  |  | Placebo |  |  | 69 | 67.0 ±14.0 | 27 (39) | 7.9 ±3.5 | 47 (68) | 35 (51) |
| Hwang  et al (ATESS) (28) | South Korea multi-centered clinical trial | Septic shock | Vitamin C (50 mg/kg, maximum single dose 3 g)+thiamine (200 mg) q12h for a total of 48 h | 8.4-9.9 h from ED arrival to the first study drug | Glucocorticoid was administered to over half of the patients (58.5% for Treatment and 50% for Placebo group) | 53 | 70 (62–76) | 20 (37.7) | 8 (6–10) | 53（100） | 12 (22.6) |
|  |  |  | Placebo |  |  | 58 | 69 (62–74) | 22 (37.9) | 8 (6–10) | 58（100） | 14 (24.1) |
| Chang  et al (HYVCTTSSS trial) (29) | China single center clinical trial | Sepsis and septic shock | Hydrocortisone (50 mg q6h for 7 days or until ICU discharge, whichever occurred first), vitamin C (1.5 g q6h for 4 days or until ICU discharge, whichever occurred first), and IV thiamine (200 mg q12h for 4 days or until ICU discharge, whichever occurred first). | Not reported | Not reported | 40 | 59.5±15.0 | 22 (57.5) | 9.6±4.5 | 22 (55) | 30 (75) |
|  |  |  | Placebo |  |  | 40 | 63.7±12.8 | 21 (52.5) | 10.1±4.0 | 24 (60) | 32 (80) |
| Fujii  et al (VITAMINS  trial) (30) | International multi-centered clinical trial | Septic shock | Vitamin C (1.5 g q6h)+hydrocortisone (50 mg q6h)+thiamine (200 mg q12h) until shock resolution or up to 10 days | Not reported | 42.1% patients in the intervention group and 37.5% of control patients received hydrocortisone | 107 | 61.9±15.9 | 68 (63.6) | 8.6 (2.7) | 107 (100) | 66 (61.7) |
|  |  |  | control group: intravenous hydrocortisone (50 mg q6h) |  |  | 104 | 61.6 ±13.9 | 65 (62.5) | 8.4 (2.7) | 104 (100) | 65 (62.5) |
| Mohamed  et al (ViCTOR trial) (33) | India single center clinical trial | Septic shock | vitamin C (1.5 g q6h)+thiamine (200 mg q12h), and hydrocortisone (50 mg q6h). The treatment was continued for at least 4 days. | Within 6h of  onset of septic shock admission | Not reported | 45 | 58.69 ± 14.89 | 31 (69) | 11.22±2.99 | 45 (100) | 22 (48.8) |
|  |  |  | control group: standard of care for septic shock |  |  | 43 | 59.37 ± 15.01 | 32 (74) | 10.89 ± 3.82 | 43 (100) | 20 (46.5) |
| Moskowitz  et al (ACTS trial) (34) | America multi-centered clinical trial | Septic shock | Ascorbic acid (1500 mg)+hydrocortisone (50 mg)+thiamine (100 mg) q6h for 4 days or until ICU discharge | From vasopressor initiation to first study drug:14.5 (8.1-19.1)h | 14 patients (14.1%) in the control group received open-label corticosteroids | 101 | 68.9±15.0 | 57 (56.4) | 9.1 ±3.5 | 101 (100) | 48 (47.5) |
|  |  |  | Placebo |  |  | 99 | 67.7±13.9 | 54 (54.6) | 9.2 ±3.2 | 99 (100) | 44 (44.4) |
| Wani  et al (31) | India single center clinical trial | Sepsis or septic shock | Vitamin C (1.5 g q6h for 4 days or until discharge from the hospital) +hydrocortisone (50 mg q6h for 7 days or until ICU discharge followed by a taper over 3 days)+thiamine  (200 mg q12h for 4 days or until discharge from the hospital) | Not reported | Not reported | 50 | 59 (25–72) | 28 (56) | 9.22±3.54 | 76.0 (38/50) | 3 (6) |
|  |  |  | standard therapy |  |  | 50 | 56 (25–72) | 31 (62) | 9.36±3.66 | 92.0 (46/50) | 3 (6) |
| Lv  et al (32) | China single center clinical trial | Sepsis | 3 g vitamin C dissolved into 5% dextrose (100 ml/time, 2 times/day) until ICU discharge | From the day of entering ICU | Not reported | 61 | 58.7±14.3 | 30 (49.2) | 8.6±2.9 | 35 (57.4) | 31 (50.8) |
|  |  |  | Control group: routine and basic therapy with intravenous drip of 5% dextrose and placebo (100 ml/time, 2 times/day) |  |  | 56 | 60.2±14.1 | 29 (51.8) | 8.9±3.1 | 33 (58.9) | 28 (50.0) |
| Fowler  et al (CITRIS-  ALI trial) (35) | America multi-centered clinical trial | sepsis and ARDS present for less than 24 hours | Vitamin C  (50 mg/kg q6h, Study drug infusion was stopped when the final dosage was administered (ie, hour 96) or at ICU discharge, discharge from the study hospital, study withdrawal, or death, whichever occurred first) | Within 6 hours of randomization or at earliest available times after clinically indicated procedures (eg, imaging) requiring patients to be out of the ICU | 67% received corticosteroid  in vitamin c group and 65% in placebo group | 84 | 54 (39–67) | 45 (54) | 9.8 (3.2) | 54 (64.3) | 84 (100) |
|  |  |  | Placebo |  |  | 83 | 57 (44–70) | 45 (54) | 10.3 (3.1) | 59 (71.1) | 83 (100) |
| Zabet  et al (36) | Iran single center clinical trial | Septic shock | Ascorbic acid (25 mg/kg q6h for 72 h) | Not reported | Not reported | 14 | 64.14±15.98 | 10 (71.42) | 11.78±2.22 | 14（100） | Not reported |
|  |  |  | Placebo |  |  | 14 | 63.71±13.84 | 11 (78.57) | 12.35±3.00 | 14（100） | Not reported |

**
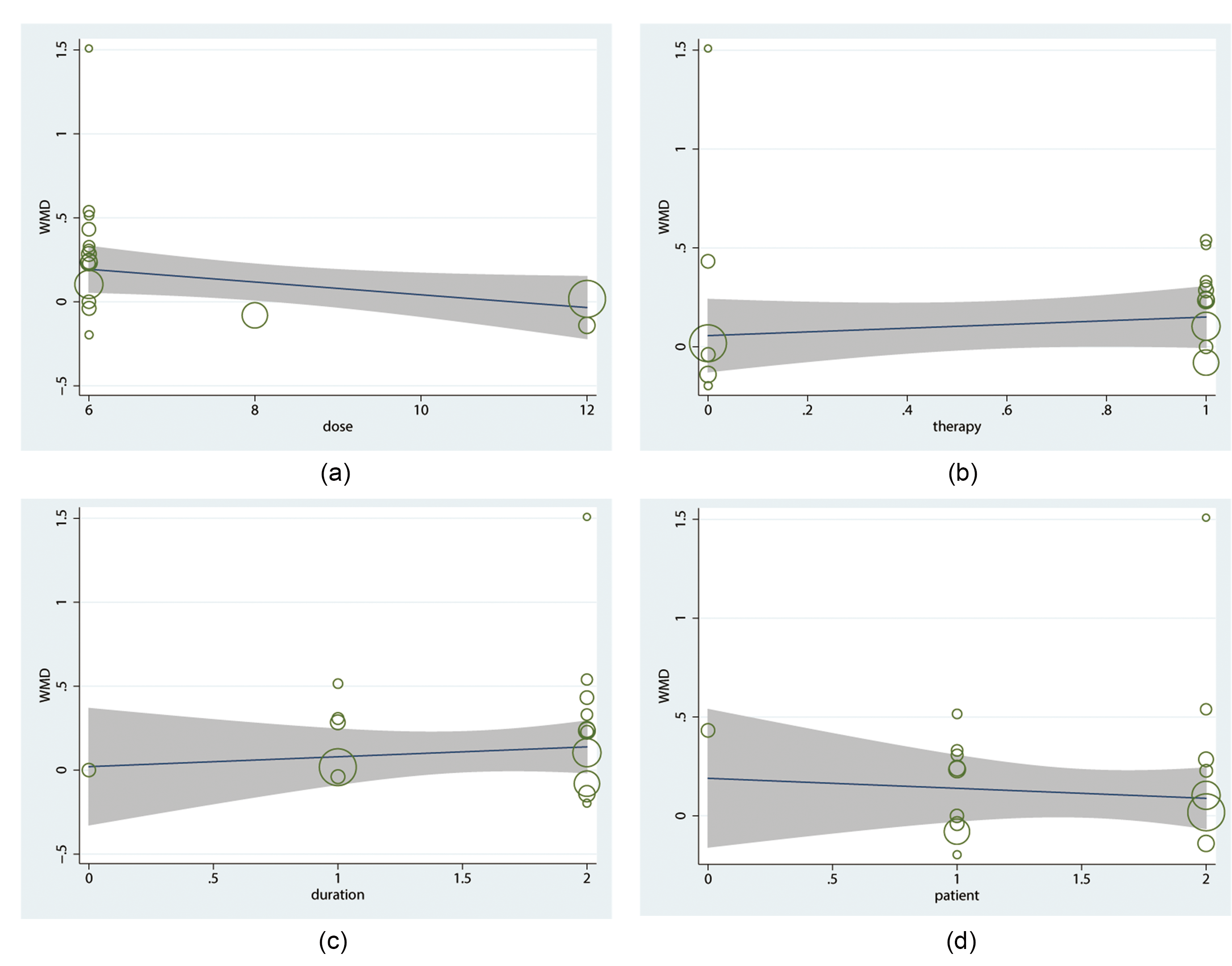
Fig. S1.** Meta-regressions analysis of delta SOFA score.

**
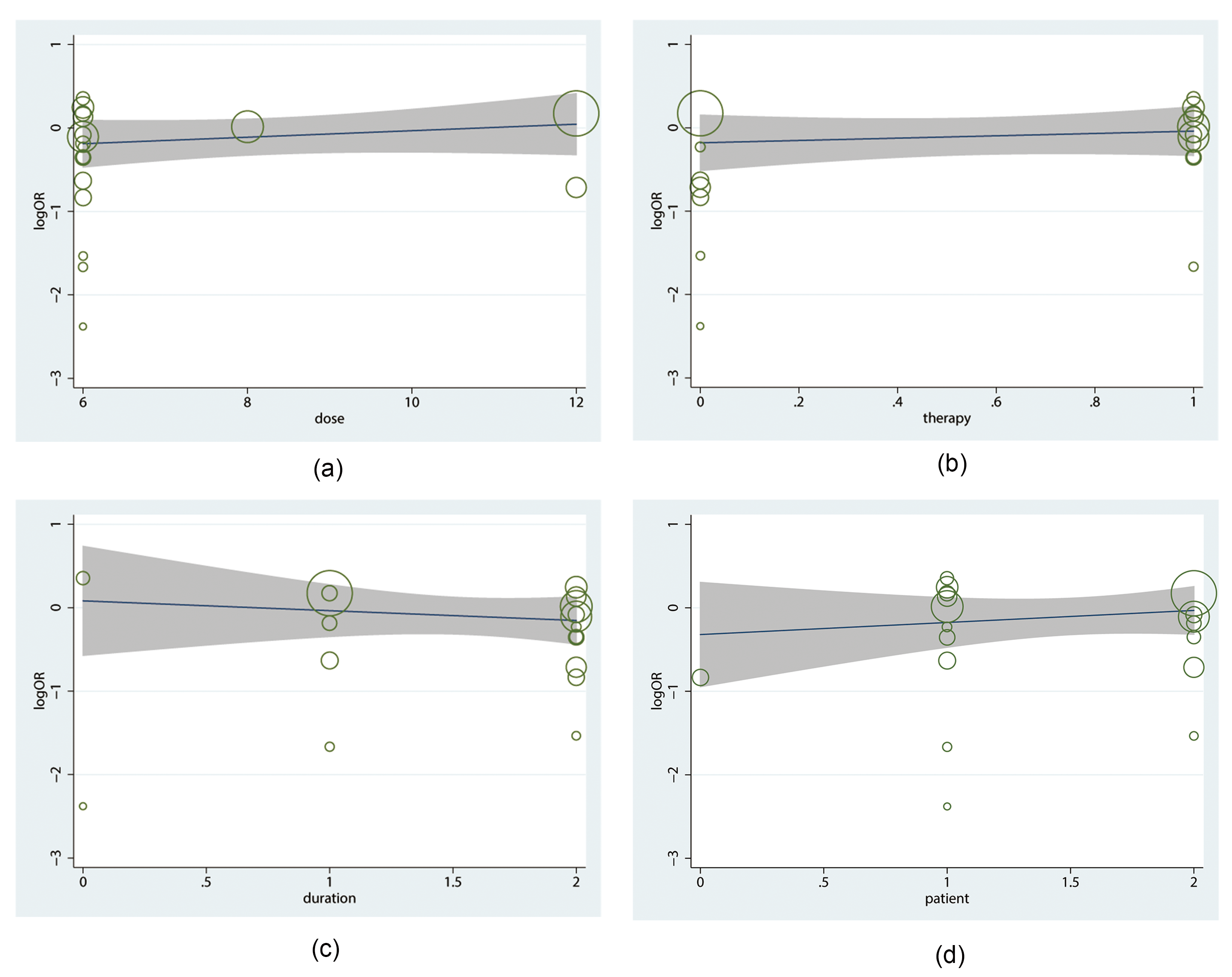
**

**Fig. S2.** Meta-regressions analysis of short-term mortality.
